# Sedimentary supply of humic-like fluorescent dissolved organic matter and its implication for chemoautotrophic microbial activity in the Izu-Ogasawara Trench

**DOI:** 10.1038/s41598-021-97774-7

**Published:** 2021-09-24

**Authors:** M. Shigemitsu, T. Yokokawa, H. Uchida, S. Kawagucci, A. Murata

**Affiliations:** 1 Physical and Chemical Oceanography Research Group, Global Ocean Observation Research Center, Research Institute for Global Change, Japan Agency for Marine-Earth Science and Technology, Yokosuka, Japan; 2 Super-Cutting-Edge Grand and Advanced Research Program, Institute for Extra-Cutting-Edge Science and Technology Avant-Garde Research, Japan Agency for Marine-Earth Science and Technology, Yokosuka, Japan

**Keywords:** Biogeochemistry, Ocean sciences

## Abstract

Microbial community structure in the hadal water is reported to be different from that in the upper abyssal water. However, the mechanism governing the difference has not been fully understood. In this study, we investigate the vertical distributions of humic-like fluorescent dissolved organic matter (FDOM_H_), chemoautotrophic production, apparent oxygen utilization (AOU), and N* in the Izu-Ogasawara Trench. In the upper abyssal waters (< 6000 m), FDOM_H_ has a significantly positive correlation with AOU; FDOM_H_ deviates from the relationship and increases with depth without involving the increment of AOU in the hadal waters. This suggests that FDOM_H_ is transferred from the sediments to the hadal waters through pore water, while the FDOM_H_ is produced in situ in the upper abyssal waters. Chemoautotrophic production and N* increases and decreases with depth in the hadal waters, respectively. This corroborates the effluxes of dissolved substances, including dissolved organic matter and electron donors from sediments, which fuels the heterotrophic/chemoautotrophic microbial communities in the hadal waters. A simple box model analysis reveals that the funnel-like trench topography facilitates the increase in dissolved substances with depth in the hadal waters, which might contribute to the unique microbiological community structure in these waters.

## Introduction

The hadal zone, at seawater depths of > 6000 m, is the last frontier of exploration and investigation on Earth due to its limited accessibility. It was generally believed that the hadal zone was oligotrophic similar to the upper abyssal zone due to a limited amount of sinking organic matter reaching the zones from the sunlit surface ocean, and that the only difference between the hadal and upper abyssal zones was the hydrostatic pressure. However, unique and active benthic microbiomes in the hadal trench sediments have now been confirmed^1,2^.

In contrast to the hadal benthic microbiological studies^1,2^, the studies on the hadal water column are limited. Recently, some intriguing aspects of the hadal water column microbiomes have been illuminated^3^. For example, the microbial community structure in the Mariana Trench has been reported to be different from that in the upper abyssal waters^3^. Specifically, the abundance of potentially chemoautotrophic populations in the hadal waters was less as compared to that in the upper abyssal waters, whereas contrasting results were obtained for potentially heterotrophic populations. The chemoautotrophic nitrifiers in the hadal waters adapted to the higher flux of electron donors (i.e., nitrite and ammonium), while those found in the upper abyssal waters adapted to the lower flux of electron donors. Based on these results, it was hypothesized that the sediment resuspension from the trench slope supplies organic matter to the hadal waters, and that the resuspended organic matter and electron donors derived from the decomposition of organic matter produced a microbial population that can only be found in the trench. Sediment resuspension in hadal waters has also been suggested in other studies. Kawagucci et al.^4^ invoked the resuspension of slope sediments to explain the heterogeneous spatial distributions of methane concentrations and isotopic compositions in the Izu-Ogasawara Trench. Gamo and Shitashima^5^ suggested that the resuspension of the bottom sediment in the Izu-Ogasawara Trench is necessary to interpret the increase in the concentrations of total dissolvable Fe and Mn with depth in the hadal waters. In addition, they suggested the efflux of silicate from the bottom sediment in the trench as a mechanism that increases the silicate concentrations in the trench with increasing depth. They also found excess ^222^Rn at depths up to 2700 m above the bottom, which indicates that the excess ^222^Rn was supplied from the trench slope as well as the bottom sediments. Because silicate and ^222^Rn are dissolved constituents and likely transferred from the sediments at the slope and bottom of the trench to the overlying waters through pore water^5^, dissolved organic matter (DOM) and electron donors are also likely to be transported from the sediments to the overlying waters. Actually, various studies suggested that DOM and electron donors such as ammonium, nitrite, and hydrogen sulfide are released from sediments to overlying waters^6,7^. These substances may affect microbial respiration in the water column^7,8^. Hiraoka et al.^1^ showed that anaerobic environments are formed in the bottom sediments of the Izu-Ogasawara Trench, and that ammonium concentrations in the sediments increase with depth. The build-up of DOM in the sediments was deduced based on the ammonium concentration. Analogous with these previous results, there is a possibility that DOM and electron donors released from the slope/bottom sediments facilitate the heterotrophic/chemoautotrophic microbial community structure specific to the hadal waters. However, little is known about the possibility of the efflux of DOM and electron donors from the sediments to the overlying waters in the hadal environment.

Accordingly, in this study, we attempt to gain insights into the efflux of DOM and electron donors from the sediments of the Izu-Ogasawara Trench. To this end, we use humic-like fluorescent dissolved organic matter (FDOM_H_) and dissolved inorganic carbon (DIC) fixation activity by chemoautotrophic microbes as tracers of DOM and electron donors that are transported from the sediments to the overlying waters, respectively. FDOM has been used as a tracer of DOM in the water column^9,10^ and seafloor sediment^11,12^. In the latter studies^11,12^, the positive correlations between FDOM_H_ and dissolved organic carbon (DOC) were confirmed. Additionally, FDOM_H_ is reported to be produced more in anoxic sediments than in suboxic or mixed sediment redox conditions^12^, while exhibiting an increased production in hypoxic conditions than in oxic ones^13^. Thus, FDOM_H_ is potentially suitable as a tracer of DOM supplied from the slope and bottom sediments in the trench. Meanwhile, DIC fixation activity is related to electron donor availability and could even be detected in the Atlantic deep waters where the concentrations of electron donors like nitrite and ammonium as possible energy sources for chemoautotrophic microbes are extremely low^14–16^. We therefore considered DIC fixation activity to be a suitable tracer of electron donors released from the sediments.

**Figure 1:**
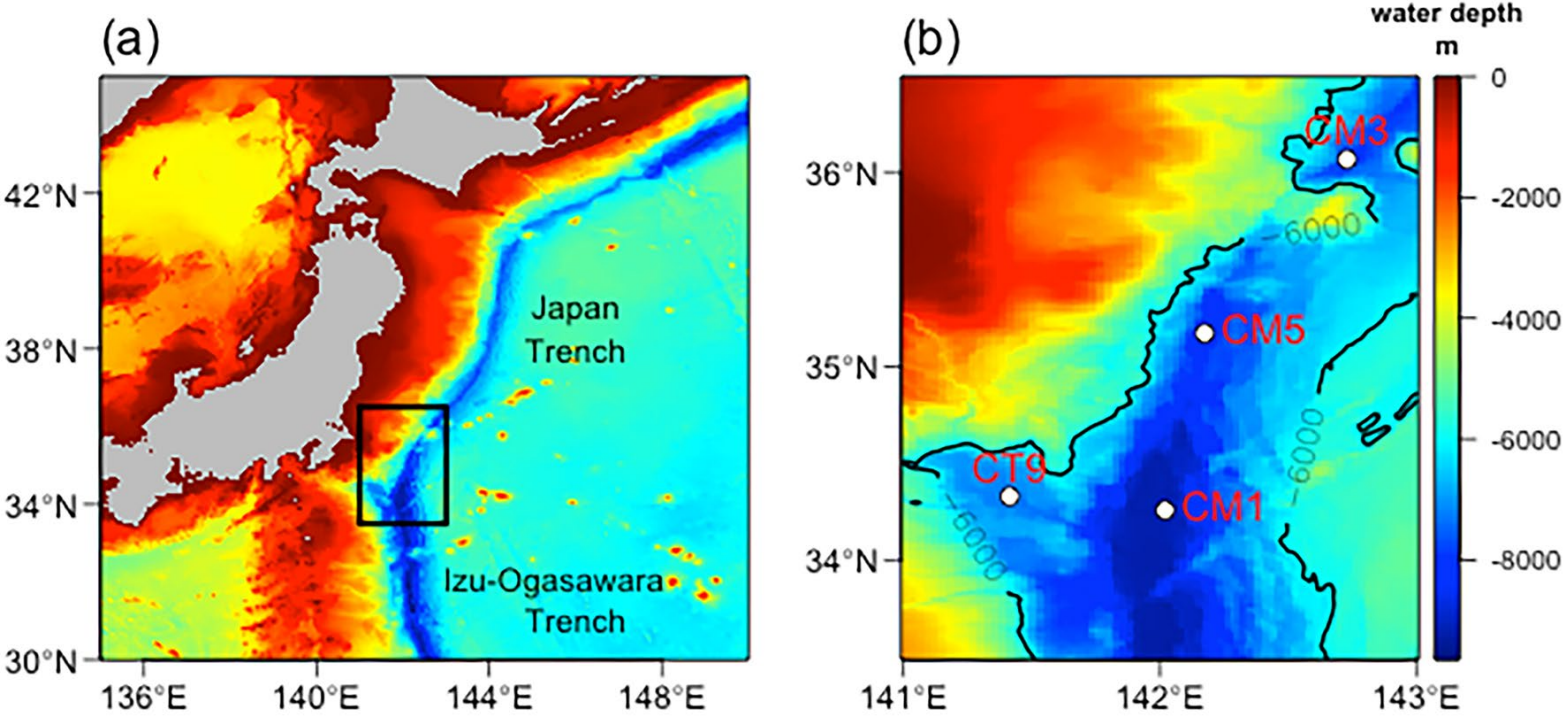
Maps of the observation area and sampling sites in this study. The area in the black rectangle in (**a**) is expanded in (**b**). CM1, CM5, and CT9 are the stations in the Izu-Ogasawara Trench, and CM3 is the station in the Japan Trench.

**Figure 2:**
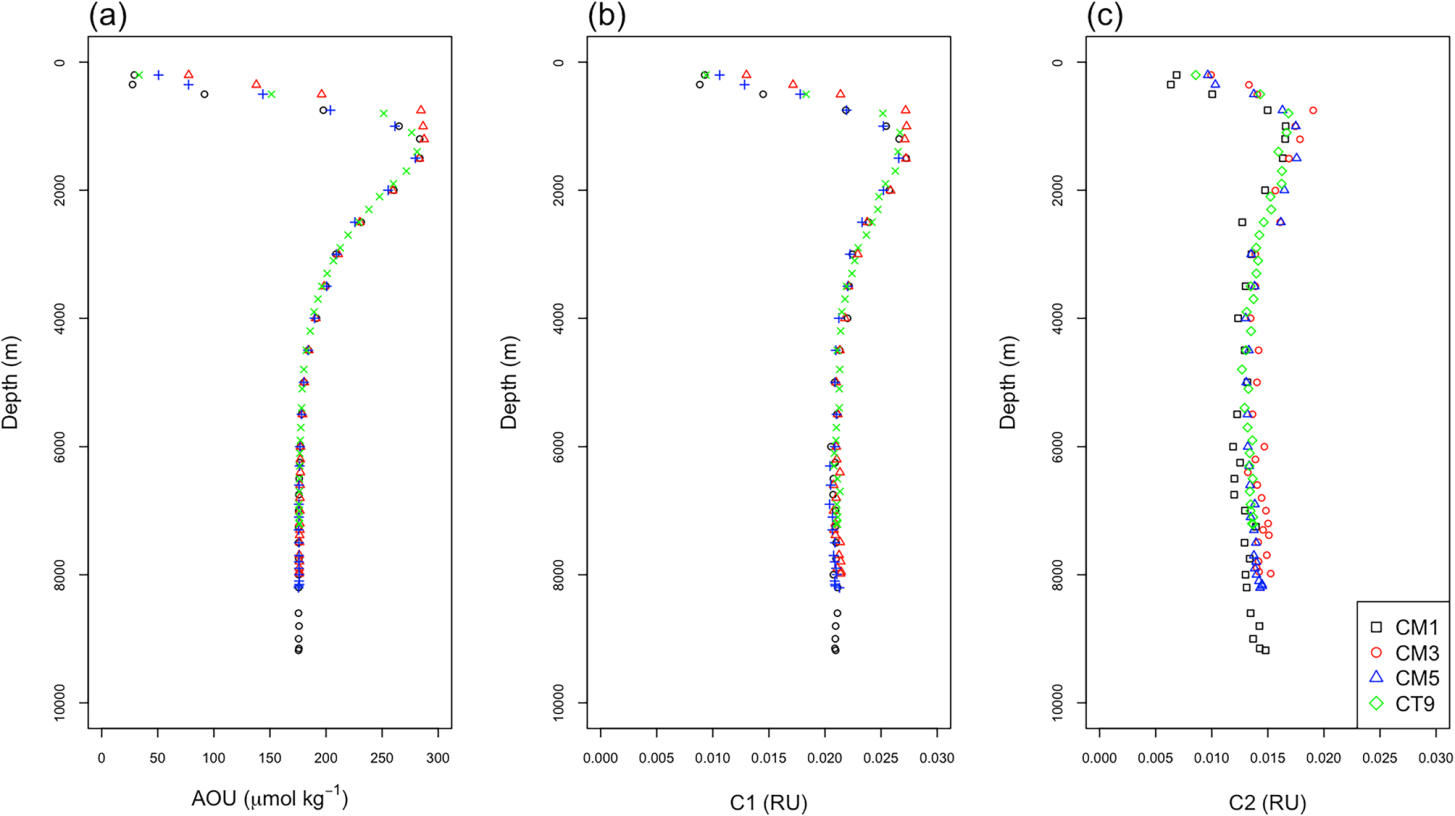
Vertical distributions of (**a**) AOU (μmol kg^−1^), humic-like FDOM (**b**) C1 (RU), and (**c**) C2 (RU). Black squares represent CM1, red circles represent CM3, blue triangles represent CM5, and green diamonds represent CT9.

The objectives of this study are to (1) detect the increase in the FDOM_H_ and DIC fixation rate with depth in the Izu-Ogasawara Trench (Fig. 1), (2) gain insights into the DOM and electron donor effluxes, and (3) propose the possibility that the effluxes of DOM and electron donors from the sediments contribute to the unique microbial community structure in the hadal waters.

## Results

### FDOM distributions in and above the trench

A three-component model was obtained (Supplementary Fig. 1) using parallel factor (PARAFAC) analyses for excitation-emission matrix (EEM) fluorescence. This model comprised two humic-like components (C1, C2) and one protein-like component (C3). C1 exhibited an excitation/emission pair of 250/467 nm for the primary peak and that of 365/467 nm for the secondary peak. In contrast, C2 demonstrated an excitation/emission pair of 250/407 nm for the primary peak and that of 335/407 nm for the secondary peak. C1 and C2 have been traditionally defined to be terrestrial and marine, respectively. The two humic-like components were identified in the northwestern Pacific, Japan Sea^17,18^, and global oceans^9^. As mentioned in the “Methods” section, based on the vertical distributions, the protein-like component (C3) was affected by contamination. Therefore, this component is not discussed hereafter.

C1 and C2 showed similar vertical distributions to those of the apparent oxygen utilization (AOU) (Figs. 2 and 4) in depths ranging from 200 to 6000 m; there were significant positive correlations between C1 and AOU, and between C2 and AOU (*r*^2^ = 0.98, *n* = 70, *p* < 0.001; and *r*^2^ = 0.86, *n* = 70, *p* < 0.001), respectively. In addition, the slope value of 6.8 × 10^–5^ for C1 was greater than that of 3.6 × 10^–5^ for C2. These results indicate that the two humic-like components are produced in the ocean interior and that C1 production rate relative to unit oxygen consumption is greater than that of C2, which is similar to the results of previous studies^9,17–21^. The difference between the production rates might be associated with a microbial community related to production of each FDOM_H_^22^. Unlike the upper abyssal waters, C1 and C2 slightly increase with depth in the hadal waters, while AOU remains relatively constant, resulting in no significant correlations between them (*r*^2^ < 0.02, *n* = 52, *p* > 0.05 for both C1 and C2).

### DIC fixation rates in and above the trench

**Figure 3:**
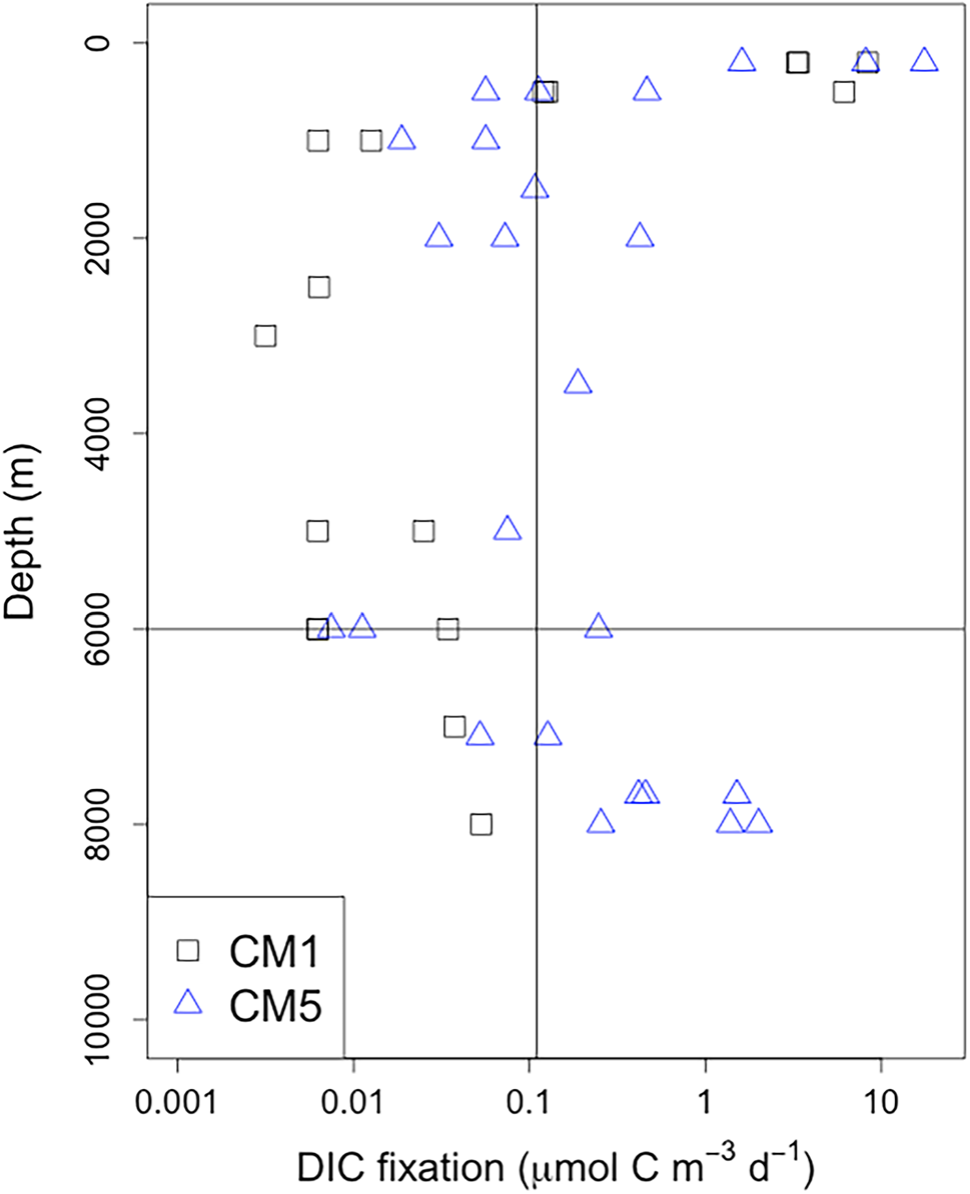
Vertical profiles of DIC fixation rate. Black squares and blue triangles represent CM1 and CM5, respectively. The horizontal black line represents a depth of 6000 m, and vertical black line represents the detection limit. The detection limit is calculated to be three times the standard deviation of the DPM of blank samples. When the negative values are calculated after the blank corrections, the values are not shown here. Horizontal axis is shown on a logarithmic scale.

DIC fixation rates at CM1 and CM5 showed similar vertical distributions to each other (Fig. 3). The DIC fixation rates for depths ranging from 200 to 500 m were high; they were relatively low for depths below 500 m to 6000 m, most of which were below the detection limit. These values and vertical distributions for depths ranging from 200 to 6000 m are similar to those found in the Atlantic Ocean^14–16^. In contrast, DIC fixation rates in the hadal waters tended to increase with depth. At CM5, DIC fixation rates in the hadal waters were higher than the detection limit.

## Discussion

### Behavior of FDOM deviated from the positive relationship between FDOM and AOU in the trench

**Figure 4:**
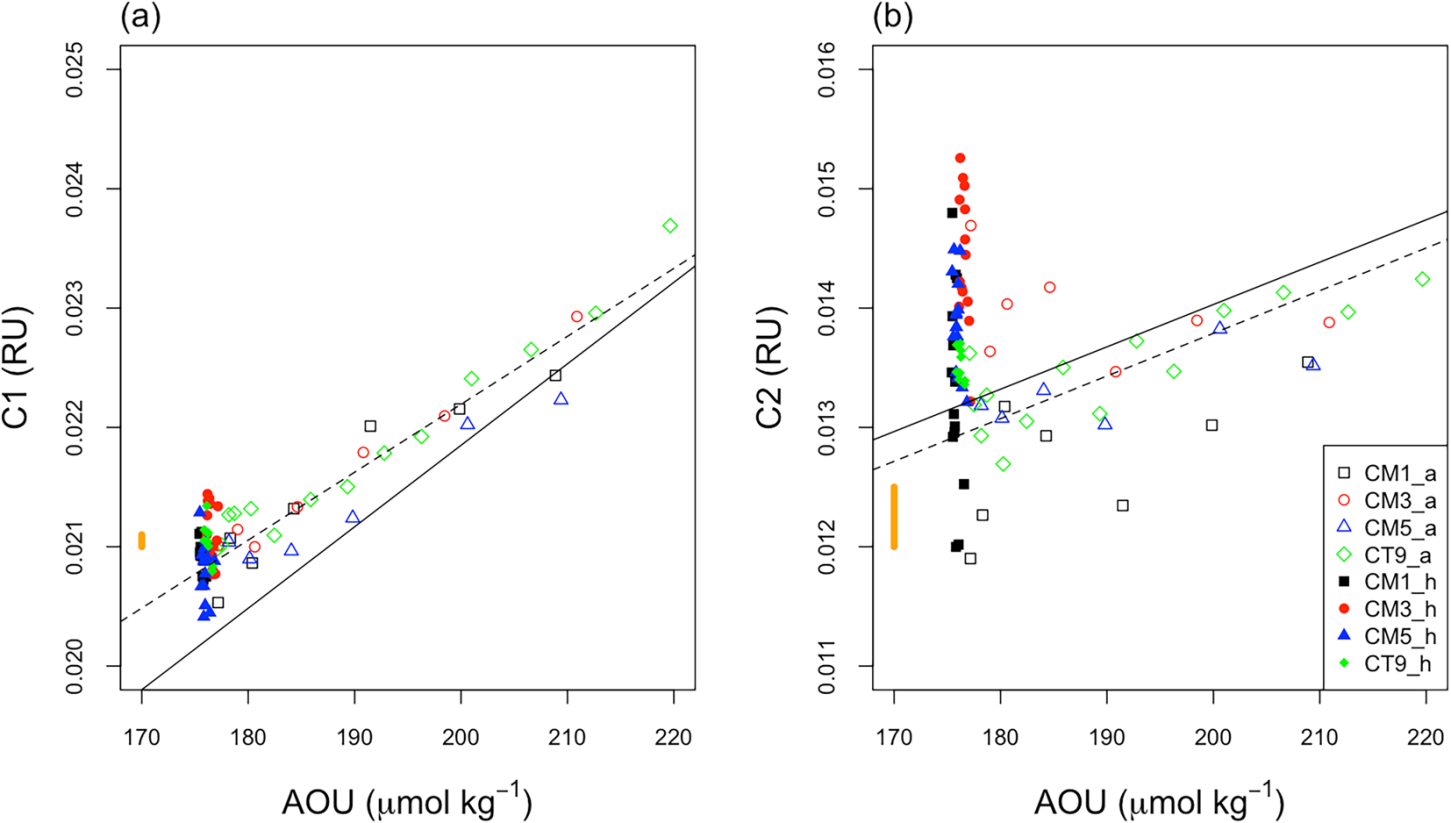
Scatter plots (**a**) between AOU (μmol kg^−1^) and humic-like FDOM C1 (RU), and (**b**) between AOU (μmol kg^−1^) and humic-like FDOM C2 (RU). The open symbols indicate the upper abyssal waters (> 2500 m), and solid symbols indicate the hadal waters. The yellow vertical line in each figure represents the measurement precision of C1 or C2, which was estimated from the standard deviation of the duplicate measurements. The black solid and dashed lines are the regression lines for the data obtained in the depth range of 200–6000 m (C1 = 0.0082 + 6.8 × 10^−5^AOU, *r*^2^ = 0.98, *p* < 0.001; C2 = 0.0069 + 3.6 × 10^−5^AOU, *r*^2^ = 0.86, *p* < 0.001 ) and 1000–6000 m (C1 = 0.0108 + 5.7 × 10^−5^AOU, *r*^2^ = 0.99 , *p* < 0.001; C2 = 0.0066 + 3.6 × 10^−5^AOU, *r*^2^ = 0.80 , *p* < 0.001), respectively.

As stated above, C1 and C2 had significantly positive correlations with AOU for depths ranging from 200 to 6000 m, respectively (Figs. 2 and 4). However, in the hadal waters, there were not significant positive correlations between C1 and AOU, and between C2 and AOU because C1 and C2 increase with depth without an increase in AOU (Figs. 2 and 4).

To explain the deviations, two mechanisms are considered: (1) particulate FDOM resuspended from the trench slope and bottom affects the increase in FDOM with depth^23^, and (2) FDOM transported from the slope and bottom sediments to the overlying waters through pore water contributes to the vertical increase^24^. In the hadal waters, there were insufficient significant positive correlations between turbidity and C1 (*r*^2^ = 0.20, *n* = 23, *p* = 0.03), and between turbidity and C2 (*r*^2^ = 0.08, *n* = 23, *p* > 0.05). However, turbidity data were only obtained for stations CM1 and CT9. A previous study has indicated that anaerobic environments develop in the bottom sediments of the trench and allow denitrification to occur^1^. If C1 and C2 are transported from the sediments through pore water, N* (NO_3_^−^—16 × PO_4_^3−^)^25^ in the hadal waters would be affected to some extent. In fact, the seawater N* tends to decrease in the deepest parts of the hadal waters at every station (Supplementary Fig. 2). Because denitrification in oxygen-rich water column is unlikely, the low N* at the bottom water is attributable to efflux of pore waters, having low N^*^, to the overlying waters. It is difficult to explain the vertical increase in C1 and C2 levels with depth by using the first mechanism, but the second mechanism provides a more promising explanation.

From these results, it is implied that dissolved substances in the sediment pore waters can be supplied to the overlying waters from the trench slope/bottom sediments without involving the re-suspension of particulate substances. Several previous studies revealed that FDOM_H_ is positively related to DOC in seafloor sediments, where both FDOM_H_ and DOC effluxes were confirmed^11,12^. Based on these previous results and those of this study, there is a possibility that the DOM supplied along with the FDOM_H_ from the sediments fuels the heterotrophic microbial communities in the hadal waters, and the possibility will be elucidated by the ongoing measurements of heterotrophic production. The electron donor ammonium, which is supplied from seafloor sediments, has been hypothesized to influence chemoautotrophic microbial activity in the overlying waters^26^, which is partially proven by the measured DIC fixation rates in this study. Consequently, the microbial community structure in the hadal waters in this trench may be different from that in the upper abyssal waters similar to the Mariana trench^3^, and the hypothesis will also be confirmed based on the ongoing phylogenetic analyses.

### Trench topographic effect on increase of dissolved constituents in hadal waters

**Figure 5:**
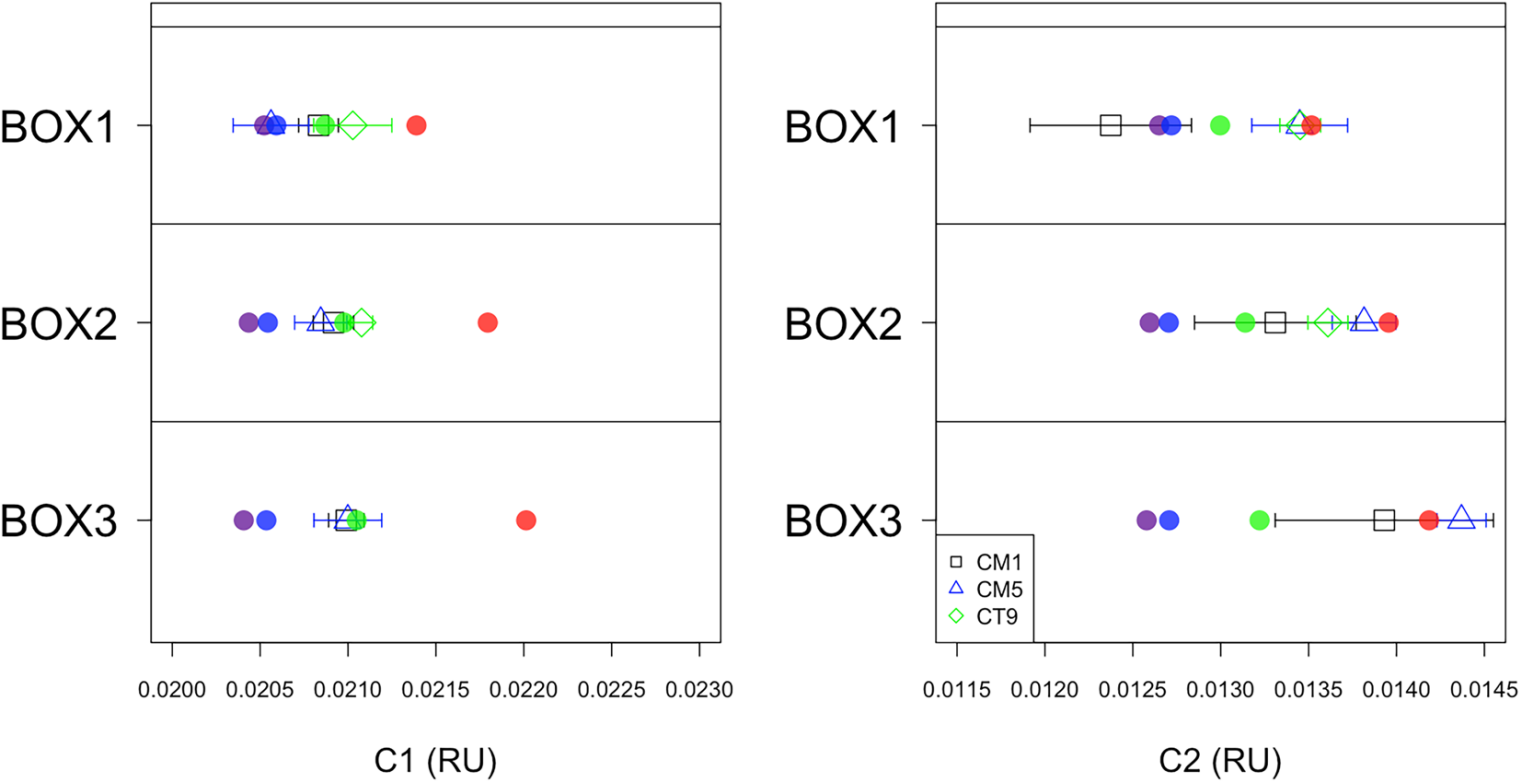
Model results for humic-like FDOM C1 (left) and C2 (right): the solid purple circle represents no flux case, solid blue circle exhibits the low flux case, solid green circles are the mid flux case, and solid red demonstrates the high flux case. The open black square with a horizontal line in each box represents the average value of humic-like FDOM measurements with a standard deviation for CM1, and the open blue triangles and open green diamonds represent CM5 and CT9.

In the Izu-Ogasawara Trench, the potential temperature and salinity are almost constant with depth (Supplementary Fig. 3), indicating that the waters inside the trench are vertically and horizontally well-mixed, similar to the results of previous studies^4,5^. Thus, we considered that physical processes affecting FDOM_H_ behavior in the trench can be expressed by a one-dimensional model. Although the biogeochemical processes related to FDOM_H_ in the trench seem to be heterogeneous, based on the spatial variations of FDOM_H_ levels in the trench (Figs. 2 and 4), a one-dimensional model can represent, to some extent, the increase in the FDOM_H_ levels with depth in the trench. Here, a simple three-box model was applied to obtain deeper insights into the increase of C1 and C2 with depth in the hadal waters. As stated in the “Methods” section, we considered physical (one-way advection and two-way mixing) and biogeochemical (sedimentary supply and decomposition) processes for FDOM_H_ in the box model. With the fixed physical processes and one biogeochemical process, i.e., decomposition, we considered four cases for the sedimentary flux of FDOM_H_: (1) no flux of C1 and C2 from the slope/bottom sediments, (2) low flux of 20 (Raman unit L; RU L) m^-2^ yr^−1^ estimated in the Arctic shelf sediment^27^, (3) mid flux of 100 (RU L) m^−2^ yr^−1^ set by considering the previous observation in the Japan Sea^24^ and (4) high flux of 250 (RU L) m^-2^ yr^−1^, based on previous observations in the eastern tropical South Pacific (ETSP) off the coast of Peru^7^ (see below). In the “no flux” case, the FDOM_H_ levels decreased with depth due to the decomposition (Fig. 5). In the “low flux” case, the prescribed flux was not sufficient to explain the increase in the FDOM_H_ with depth and the FDOM_H_ levels slightly decreased. In the “mid flux” case, the increase in C1 with depth could be well explained by the prescribed flux, while the flux was still not high enough to represent the increase for C2. In the “high flux” case, the prescribed sediment flux was too high to explain the increase with depth for C1, but was optimal for C2.

Interestingly, the increase in FDOM_H_ levels with depth could be represented by the proposed model, although we only considered the constant sediment flux in each box for both mid and high flux cases. The results could be explained based on the following characteristics of the trench: (1) Because the waters in the trench are surrounded by the slopes, waters entering the deeper part of the trench have the higher level of FDOM_H_ compared to those in the shallower part, (2) the ratio of slope area to volume is the lowest for box 1 (6.1 × 10^4^m^−1^), which is followed by that of box 2 (6.9 × 10^4^m^−1^) and box 3 (14.3 × 10^4^m^−1^), and (3) the horizontal area of the trench decreases with depth, resulting in the decline in volume transport via vertical mixing with depth (see Methods). These trench topographic effects are important to represent the increase in the FDOM_H_ levels with depth. The other dissolved substances such as silicate and ^222^Rn also showed an increase with depth in the Izu-Ogasawara Trench^5^, and the same mechanism may, to some extent, contribute to the increase with depth even though the sediment flux would be vertically and spatially heterogeneous in the trench. Similarly, electron donors such as nitrite and ammonium may be increased by the topographic effect, which has been inferred from the increase in the DIC fixation rates with depth in the hadal waters.

The sediment fluxes used in the low, mid and high flux cases were determined in the following way. The flux value of 20 (RU L) m^−2^ yr^−1^ in the low flux case was adopted from the Arctic shelf study^27^. The ammonium concentrations, a proxy of organic matter decomposition, in the Arctic shelf sediment increased to ~ 200 μmol L^−1^ with depth in the upper 30 cm. Sinking fluxes of particulate organic matter produced in sunlit ocean surfaces generally decline with depth^28^, and we assume that the benthic decomposition rate of organic matter in the Arctic shelf sediment is much higher than that in the slope/bottom sediments of the Izu-Ogasawara Trench because the amount of organic matter reaching the Arctic shelf sediment should be higher than that in the Izu-Ogasawara Trench from the viewpoint of the varying bottom depths. However, the ammonium concentrations in the bottom sediments of the trench at depths in the upper several centimeters increased to the same order of magnitude as the Arctic shelf sediment^1^. This would be maintained by the relatively high sedimentation rate in the trench environment^2^ and the high TOC concentrations (0.5–6%) in the sediments of Izu-Ogasawara Trench^1,29^. Thus, we considered that the flux value of 20 (RU L) m^-2^ yr^-1^ would be appropriate for this study. However, the flux value was too small to reconstruct the observations. Therefore, the flux values of 100 and 250 (RU L) m^−2^ yr^−1^ were also used by considering the previously estimated values in the Japan Sea^24^ and ETSP off Peru^7^. The study in the Japan Sea estimated the flux of C1 and C2 from the bottom sediment to be about 1 × 10^12^ quinine sulfate unit (QSU) m^3^ yr^−1^. We converted the value to ~ 100 (RU L) m^−2^ yr^−1^ by using the area of Japan Sea (933,000 km^2^)^30^ and the relationship between QSU and RU (RU = 0.0767 × QSU)^31^ in the following way: 0.0767 × 1 × 10^12^ × 10^3^/(933,000 × 10^6^) =  ~ 82 (RU L) m^−2^ yr^−1^. Thus, the value of 100 (RU L) m^-2^ yr^−1^ was used in the mid flux case, but the value was found not to be sufficient to reconstruct the observations of C2 in trial and error of the model runs. In the study on the ETSP, much higher fluxes of C1 and C2 were observed (~ 200–15,000 (RU L) m^-2^ yr^−1^)^7^ because the sediments were retrieved closer to the land compared to those in the Japan Sea study^24^. To obtain a better result for C2, we used the flux value of 250 (RU L) m^-2^ yr^−1^ in the high flux case. As stated above, the organic matter contents in the Izu-Ogasawara Trench range from 0.5 to 6%^1,29^, while those in the Japan Sea and ETSP off the coast of Peru are between 1 and 5%^24^ and between 3 and 14%^32^, respectively. The reason as to why the flux values of 100 and 250 estimated to be optimal in this study are close to the flux value obtained in the Japan Sea is probably because the organic carbon contents in the Izu-Ogasawara and Japan Sea sediments are similar. Consequently, the sediment fluxes for C1 and C2 were estimated to be ~ 100 (RU L) m^-2^ yr^−1^ and be ~ 250 (RU L) m^−2^ yr^−1^, respectively. These results were within the previous results^7,24,27^. Differently from this study, the sediment fluxes of C1 and C2 were similar to each other or those for C1 were greater than those for C2 in previous studies^7,24,27^. Obviously, the reason why the sediment flux of C2 was higher than that of C1 needs to be further investigated.

There are two caveats in this study. One is that we measured the FDOM_H_ levels for the unfiltered seawater samples and particulate FDOM_H_ might be included in the measurements although there were not significant positive correlations between FDOM_H_ and turbidity in the hadal waters. Thus, for now, we cannot completely exclude the possibility that particulate FDOM_H_ derived from the resuspension of the slope sediments, to some extent, contributed to the vertical increase in the trench and the sedimentary fluxes of C1 and C2. The other caveat is that other possible mechanisms contributing to the increase in FDOM_H_ with depth in the Izu-Ogasawara Trench may exist. In this study, we clarified that the steady sedimentary flux of FDOM_H_ within the previously reported range^7,24,27^ can explain our observations. Previous studies in the Izu-Ogasawara Trench hypothesized that dissolved and particulate substances could be intermittently released and suspended from sediments in the hadal zones by episodic phenomena, such as earthquakes and/or submarine volcano eruptions^4,5^. The current study is based on a one-time observation, and the previous occurrence of episodic phenomena cannot be ruled out, i.e., episodic-event-induced suspended particles could have already settled and event-induced dissolved substances could have remained in the trench water column when we conducted our observation. Thus, further investigation is required to differentiate steady from episodic sedimentary supply.

In a previous study, the microbial cell abundance and carbon turnover rates in the bottom sediments of the Mariana Trench were found to be higher as compared to those in the adjacent abyssal plain sediments^2^. The higher microbial cell abundance and carbon turnover rates are maintained by the higher accumulation rate of the sedimentary organic matter in the hadal sediments than in the adjacent abyssal plain^2^. The higher accumulation of sedimentary organic matter is reported to be driven by landslides and funnel-like trench topography^33,34^. Thus, the mechanisms caused by trench topography, which govern the high accumulation rate of organic matter^2^ and facilitate the increase in dissolved substances with depth (this study), are deemed important for constructing microbial community structures specific to hadal waters.

## Conclusion

We examine FDOM_H_, chemoautotrophic production, AOU, and N* in the Izu-Ogasawara Trench to gain an insight into the mechanisms that drive the difference in the microbial community structure in the hadal waters compared to that in the upper abyssal waters. We determine that there is a significant positive correlation between FDOM_H_ and AOU in the upper abyssal waters, while there is no such correlation (increase in FDOM_H_ with constant AOU) in the hadal waters. The positive correlation indicates that the FDOM_H_ is produced in situ in the abyssal waters, which is similar to previous studies^9,17–21^. Meanwhile, the absence of a correlation indicates a mechanism that is specific to the hadal waters, which supplies FDOM_H_ without increasing AOU. We propose the efflux of FDOM_H_ from the slope/bottom sediments as a feasible mechanism. The proposed mechanism is consistent with the observed increase in chemoautotrophic production, as a tracer of efflux of electron donors, with depth and the decrease in N* in the hadal waters.

Our box model analysis suggests that the funnel-like trench topography helps in increasing the concentrations of dissolved substances that are supplied from the sediments. This topographic effect probably contributes to the development of the microbial community structure specific to the hadal waters. The ongoing heterotrophic production and phylogenetic analyses will corroborate the proposed mechanism.

## Methods

In this study, we collected sea water samples and analyzed their FDOM, dissolved oxygen, and nutrient concentrations using 12L Niskin bottles mounted on the CTD system at three stations (CM1, CM5, and CT9) in the northern part of the Izu-Ogasawara Trench and at one station (CM3) in the Japan Trench during the R/V Kaimei cruise (KM19-04) from September 1, 2019 to September 10, 2019 (Fig. 1). The samples used for determining the DIC fixation rate were obtained by using the Niskin bottles at only two stations (CM1 and CM5) due to the incubation time constraint.

### FDOM

The samples used for elucidating FDOM were collected at depths ranging from 200 m to a depth of 10 m above the bottom. Each sample was directly taken from a spigot of the Niskin bottle and placed in pre-combusted glass vials with acid-washed Teflon-lined caps after being rinsed thrice. The samples were stored in the dark in a refrigerator until the analysis.

Spectra of EEM fluorescence were measured on board using a benchtop fluorometer (Aqualog, Horiba Scientific) after the samples were acclimated to laboratory temperature in the dark. The measurements were carried out within 24 h after water sampling. Emission scans from 248 to 829 nm were obtained at 2.33 nm intervals for performing sequential excitation from 240 to 560 nm at 5 nm intervals by using an integration time of 12 s and employing the high charge-coupled device (CCD) gain mode. Absorbance spectra were simultaneously obtained using the benchtop fluorometer. Blank subtraction and normalization of fluorescence intensities to RU^31^ were carried out as post-measurement steps. The inner filter effect correction was not applied because the absorption coefficients at 250 nm of seawater samples in this study were much lower (1.13 ± 0.31 m^−1^) than the threshold for correction (10 m^−1^)^35^. The spectra of Milli-Q water were determined on days when the EEM measurements were performed. These spectra were used for the blank subtraction and Raman normalization.

EEMs obtained were analyzed using PARAFAC analysis and PARAFAC modelling was performed using the “eem_parafac” function (R package staRdom). Contamination from protein-like fluorophores was confirmed and the excitation and emission ranges of 250 nm to 450 nm and 350 nm to 520 nm were used for the PARAFAC analysis, respectively; this was similar to the approach adopted by Tanaka et al.^17^.

To assess the effect of contamination on C1 and C2 levels, we used the values previously measured for the sea water samples collected at depths ranging from 3500 to 6000 m from July 19 to August 10, 2018 at station KEO (32°N, 144°E), S1(40°N, 158°E), and S3 (30°N, 156°E) aboard the R/V Mirai (MR18-04). These samples were also obtained using the approach mentioned above and were frozen in the dark until the analysis. The samples were then thawed, acclimated to a laboratory temperature, and measured as described above. C1 and C2 ranged from 0.0193 to 0.0246 and 0.0119 to 0.0140, respectively, which are largely covered by the ranges observed for C1 (0.0205–0.0220) and C2 (0.0119–0.0147) in this study. Thus, the effect of contamination on C1 and C2 levels can be ignored, which is similar to the previous results^17^.

### DIC fixation rate

The samples used to determine the DIC fixation rate were collected at depths ranging from 200 to ~ 8000 m. ^14^C-bicarbonate with an activity of 3,700 kBq was added to 50 mL of a seawater sample. Triplicate samples and a glutaraldehyde-fixed blank were incubated in the dark at an in situ temperature for 72 h. Incubations were stopped by adding glutaraldehyde (2% final concentration) to the samples, which was followed by filtration using 0.2-μm polycarbonate filters. The filters were stored in a refrigerator until the analysis.

In a laboratory on land, the filters were exposed to concentrated HCl fume for 12 h and dried. Subsequently, 1 mL of a scintillation cocktail (Filter-Count, PerkinElmer) was added to each filter. After about 24 h, the filters were counted in a liquid scintillation counter (TRI-CARB 4810TR 110 V, PerkinElmer). The disintegrations per minute (DPM) of the samples were corrected for the corresponding DPM of the blanks, which was followed by their conversion into the organic carbon fixation rate over the incubation time using the natural DIC concentrations measured by a coulometer.

### Dissolved oxygen and nutrients

The samples for dissolved oxygen and nutrients (nitrate and phosphate) were collected from the surface to a depth of 10 m above the bottom. Dissolved oxygen was measured onboard using the Winkler titration method^36^. A precise estimate was derived using the standard deviation of duplicate measurements (within 0.12 μmol kg^−1^). Nitrate and phosphate were determined onboard using an autoanalyzer (Quattro 2-HR, BLTEC). The precisions for nitrate and phosphate are within 0.09 and 0.01 μmol kg^−1^, respectively.

### Box model

To analyze the behavior of FDOM_H_ in the Izu-Ogasawara Trench, we used a simple 3-box model. The Izu-Ogasawara Trench was categorized into three boxes: box 1, box 2, and box 3, which included regions at depths ranging from 6000 to 7000 m, 7000 to 8000 m, and 8000 m to the trench bottom, respectively (Supplementary Fig. 4). The interface areas between the upper abyssal waters and box 1, box 1 and box 2, box 2 and box 3, and box 1 and the Japan Trench were estimated using the ETOPO 1 model released by the National Oceanic and Atmospheric Administration along with the volume of each box. In each box *i*, the following factors were considered: one-way advective flux (ADV), two-way mixing flux (MIX), sediment flux (SED), and flux caused by the decomposition of FDOM_H_ (BGC). These processes are expressed using a tracer conservation equation:

1 $$\frac{{dC_{i} }}{dt} = {\text{ADV}} + {\text{MIX + SED + BGC}}$$

where *C*_*i*_ represents the C1 or C2 level in each box *i*.

ADV and MIX read:

2 $${\text{ADV,MIX}}\left( {C_{i} } \right) = \mathop \sum \limits_{j} {\text{MF}}_{ij} /V_{i} \cdot \left( {C_{j} - C_{i} } \right)$$

where MF_*ij*_ is the volume transport between box *i* and neighboring box *j*, *V*_*i*_ is the volume of box *i*. MF_*ij*_ of ADV was estimated to be 0.87 Sv using the volume of the Izu-Ogasawara Trench (below 6000 m) derived from the ETOPO 1 model, as well as the renewal time of ~ 5 years^37^. MF_*ij*_ of MIX was estimated using a similar approach to that adopted by Lane et al.^38^. Generally, eddy-mixing coefficient multiplied by the interface area of boxes is divided by the distance between box centers. Here, we used a horizontal eddy-mixing coefficient of 2 × 10^3^ m^2^ s^−1^, which is typical for ocean global circulation models, while employing a vertical eddy-mixing coefficient of 1 × 10^–4^ m^2^ s^−1^ ^39^. The FDOM_H_ decomposition rate, *k*, in BGC was estimated using the turnover time of ~ 500 years (median values of 400 to 600 years) for FDOM_H_^9,21^, thereby resulting in the following expression: BGC = − *k*[FDOM_H_], where [FDOM_H_] represents the C1 or C2 level. The model was run until it reached a steady state and the steady state results were used for the analysis.

## Supplementary Information

Supplementary Information.
