# Supplementary material for: Sedimentary supply of humic-like fluorescent dissolved organic matter and its implication for chemoautotrophic microbial activity in the Izu-Ogasawara Trench
Source: Sci Rep. 2021 Sep 24;11:19006. doi: 10.1038/s41598-021-97774-7 (PMC8463680; doi:10.1038/s41598-021-97774-7)
Supplement: Supplementary file 1 — Supplementary Information. [file 41598_2021_97774_MOESM1_ESM.pdf]

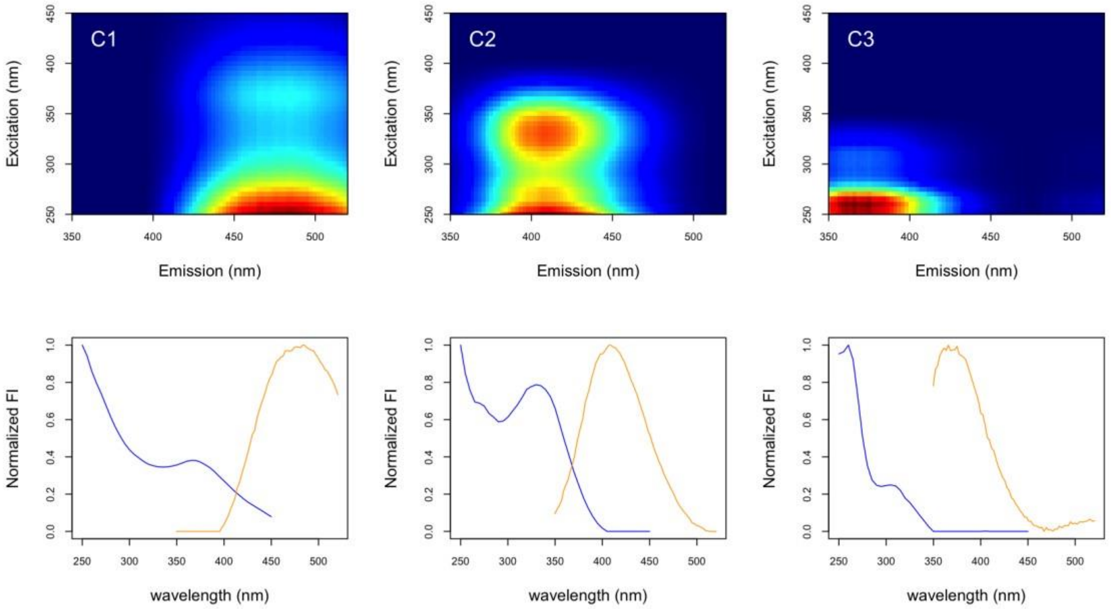

Supplementary Figure 1 Excitation-emission matrix (upper panel) and normalized fluorescence intensities (lower panels) of the three-component model determined using the PARAFAC analysis (C1, C2, and C3). The blue lines represent excitation, and orange lines indicate emission.

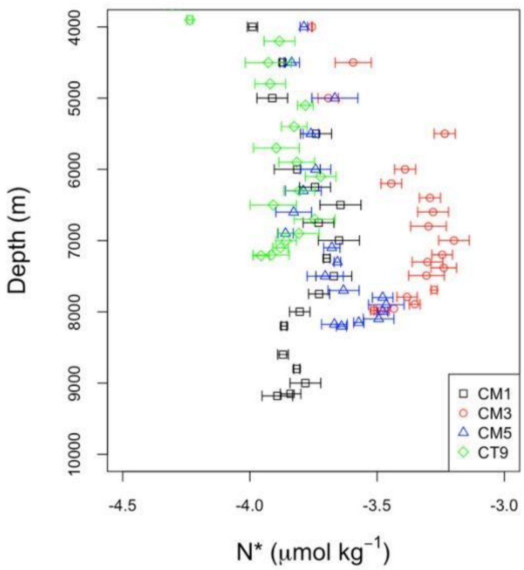

Supplementary Figure 2 Vertical distribution of N^{*}. Black squares represent CM1, red circles represent CM3, blue triangles represent CM5, and green diamonds represent CT9.

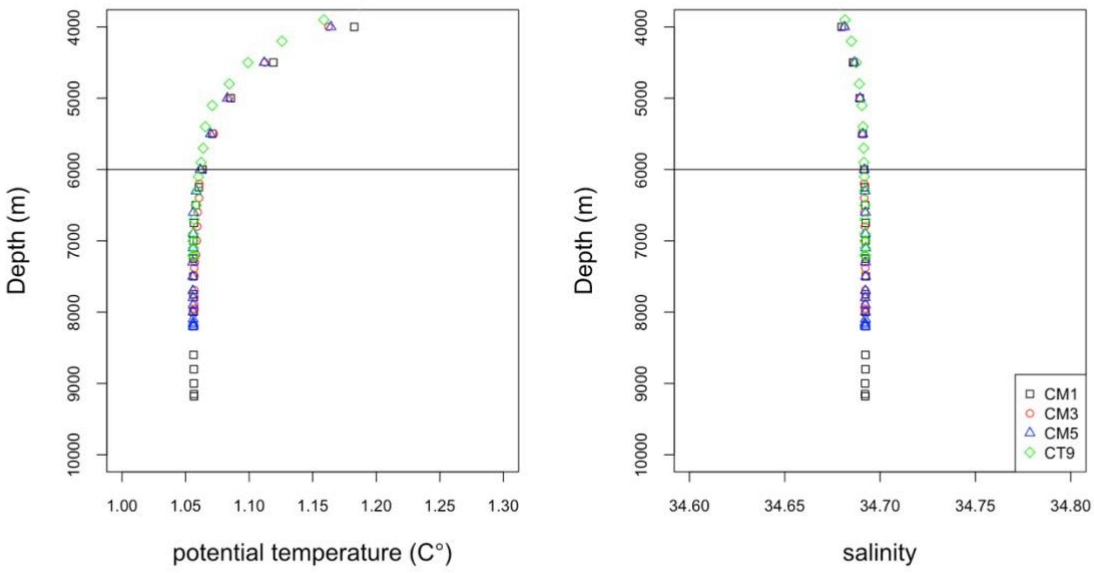

Supplementary Figure 3 Vertical profiles of a) potential temperature and b) salinity. Horizonal black line represents 6000 m depth. Black squares represent CM1, red circles represent CM3, blue triangles represent CM5, and green diamonds represent CT9.

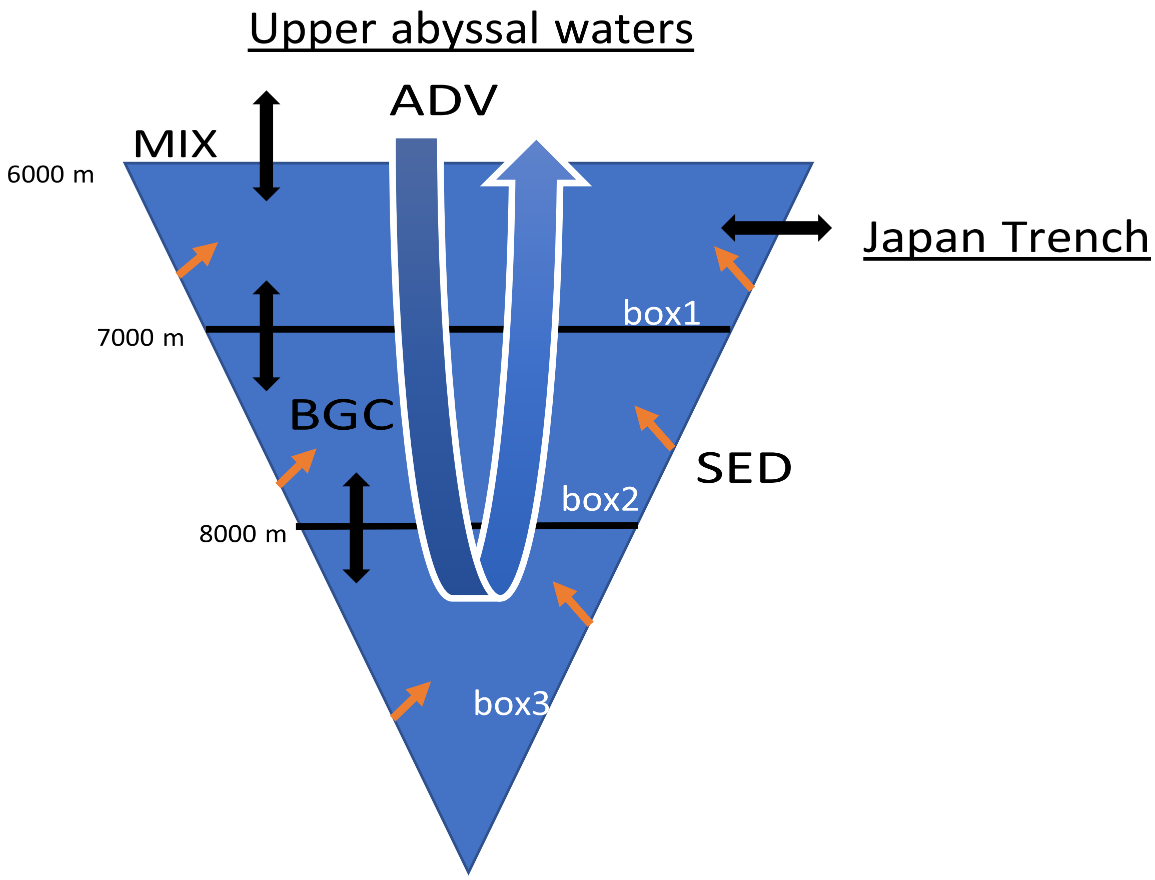

Supplementary Figure 4 Schematic of the box model used for FDOM analysis, with a representation of physical and biological fluxes. The single blue arrow represents oneway (advection) flux (ADV), whereas black double arrows show two-way (mixing) fluxes (MIX). Orange single arrows are the fluxes supplied from sediment (SED), and BGC is the fluxes obtained by the decomposition of FDOM.
